# Allergy Testing and Drug Screening on an ITO-Coated Lab-on-a-Disc

**DOI:** 10.3390/mi7030038

**Published:** 2016-02-27

**Authors:** Ho Chin Kwok, Pui Man Lau, Shu Yuen Wu, Ho Pui HO, Minghui Gao, Yiu Wa Kwan, Chun Kwok Wong, Siu Kai Kong

**Affiliations:** 1 Department of Electronic Engineering, Center for Advanced Research in Photonics, The Chinese University of Hong Kong, Shatin, Hong Kong, China; hckwok@ee.cuhk.edu.hk (H.C.K.); sywu@ee.cuhk.edu.hk (S.Y.W.); hpho@ee.cuhk.edu.hk (H.P.H.); 2 Programme of Biochemistry, School of Life Sciences, The Chinese University of Hong Kong, Shatin, Hong Kong, China; irenelau@cuhk.edu.hk (P.M.L.); minghuigao0511@gmail.com (M.G.); 3 School of Biomedical Sciences, The Chinese University of Hong Kong, Shatin, Hong Kong, China; yiuwakwan@cuhk.edu.hk; 4 Department of Chemical Pathology, Prince of Wales Hospital, The Chinese University of Hong Kong, Shatin, Hong Kong, China; ck-wong@cuhk.edu.hk; 5 Institute of Chinese Medicine and State Key Laboratory of Phytochemistry and Plant Resources in West China, The Chinese University of Hong Kong, Shatin, Hong Kong, China

**Keywords:** acridine orange (AO), basophil activation test (BAT), bisphenol A (BPA), *N*-formyl-methionine-leucine-phenylalanine (fMLP), indium tin oxide (ITO), lab-on-a-disc (LOAD), protein kinase C (PKC), phorbol 12-myristate 13-acetate (PMA), Traditional Chinese Medicine (TCM), World Allergy Organization (WAO)

## Abstract

A lab-on-a-disc (LOAD) is a centrifugal microfluidic set-up based on centrifugal force without using micro-pumps to drive reagents and cells to various chambers through channels and valves for reactions. A LOAD coated with conductive transparent indium tin oxide (ITO) for thermal control was developed to screen allergy-blocking agents. When the acridine orange (AO)-loaded KU-812 human basophilic cells were activated in the LOAD by stimuli, AO trapped in the cytoplasmic granules was released externally as an allergic mediator mimetic to report degranulation. This response was monitored by fluorescence when the released AO in supernatant had been transferred, with a higher spinning speed, from the reaction chamber to detection chamber in the LOAD where AO reacted with exogenous DNA. We report here the principles of the system and an improved LOAD set-up with the ITO-coated glass resistive microheater to run assays at 37 °C. By using this platform, we demonstrate here for the first time that triptolide, an active ingredient from the Chinese medicine herb *Tripterygium wilfordii* Hook f.*,* was able to suppress the fMLP-mediated degranulation in basophils. This serves as an example how LOADs can be used to screen agents to alleviate symptoms of allergy.

## 1. Introduction

Allergy occurs when a person’s immune system mounts an abnormal response with IgE antibody to a normally harmless substance, which is referred to as an allergen. According to the WAO (World Allergy Organization) White Book on Allergy: Update 2013, allergic diseases have now become a major global public health issue. It is estimated that more than 300 million people suffer from asthma and food allergies [1]. For the patients with allergy, avoiding causal allergens is still the best way to prevent and control allergic disease. The identification of allergens has therefore been an important step in allergy diagnosis, treatment, and management [2].

To identify allergens, the current practice is to introduce suspected allergens into the skin of patients using needles or lancets to trigger allergic reactions. Obviously, this skin-prick-test is infuriating and sometimes life-threatening [3]. Over the years, many immunoassays and diagnostic kits have been developed to quantify the level of IgE in a patient’s serum and to identify the antigens that patients are allergic to. One of the drawbacks of this approach is that positive results from these tests do not necessarily imply that the allergens are able to cross-link their corresponding IgE antibodies bound to the membrane Fc receptors of mast cells or basophils for the release of allergic mediators [4]. Additionally, more and more non-IgE type allergic cases were reported as a result of contact with xenobiotics without the involvement of IgE [5,6,7,8]. For example, xenoestrogen bisphenol A (BPA) is a well-known non-IgE type allergic trigger nowadays [9].

To this end, a functional cell-based assay, known as the basophil activation test (BAT), has been developed to assess both the IgE and non-IgE type allergy through the measurement of released allergic mediators such as histamine or beta*-*hexosaminidase or the expression of markers CD63 and CD203c on cell surface [10]. The beauty of the BAT is that basophils can be isolated from blood more easily than mast cells in tissues. Additionally, apart from the diagnostic purposes [11,12], the BAT can be used to screen mimotopes (peptides which mimic the structure of an epitope) or other natural products for immunotherapy to treat allergy [13]. Yet basophils represent only 1% of the circulating white blood cells, and the reactivity of basophils starts to decline four hours after blood taking [14]. To avoid this cumbersome, time-consuming and costly basophils purification step, cell lines such as human basophilic KU-812 cells can be used as a substitute [15]. In this paper, we demonstrate an integration platform called lab-on-a-disc (LOAD), a CD-like centrifugal microfluidic setup, which works with the KU-812 cells for the BAT to screen agents that can block allergic activities.

A LOAD is a centrifugal microfluidic set-up based on centrifugal force to drive reagents to various chambers or compartments for reactions without using micro-pump for liquid handling. Flow control is achieved by varying the spinning speed, spinning direction, and operations such as valve opening, siphoning, volume metering, mixing, and flow switching. In our previous studies, a LOAD platform was built for allergy testing [16,17]. In our second generation LOAD, the AO-loaded cells that were sensitized with a patient’s serum or medium alone were challenged with suspected allergens or other triggers in chamber at body temperature (37 °C). Fluorescence of the AO released from cellular granules was determined in the detection chamber with DNA in solution. In this paper, we demonstrated that a power-enabled LOAD coated with indium tin oxide (ITO) retains a temperature of 37 °C during reactions. This LOAD platform also offered many other advantages such as short analysis time, enhanced sensitivity, simplified procedures, low cost, and minimal consumption of samples and reagents. In this study, we highlight the modifications in the next generation LOAD and demonstrate how the LOAD system could be used to screen agents to alleviate symptoms of allergy.

## 2. Materials and Methods

### 2.1. Reagents

The polydimethylsiloxane (PDMS, Sylgards184) was from Dow Corning, Midland, MI, USA. Human KU-812 basophils and HepG2 cells were obtained from ATCC (The American Type Culture Collection, Manassas, VA, USA). Fetal bovine serum (FBS) was purchased from Gibco, Waltham, MA, USA. The RPMI 1640 and phenol red-free RPMI 1640 (pRPMI) media and penicillin/streptomycin (PS) were purchased from Invitrogen, Carlsbad, CA, USA. Acridine Orange (AO), calf thymus DNA, triptolide, phorbol 12-myristate 13-acetate (PMA) and *N*-formyl-methionine-leucine-phenylalanine (fMLP) were purchased from Sigma-Aldrich, St. Louis, MO, USA, while ionomycin was obtained from Merck, Boston, MA, USA.

### 2.2. Cell Culture and Imaging

KU-812 cells were cultured in a complete RPMI 1640 medium supplemented with 10% FBS and 1% of PS at 37 °C, 5% CO_2_. To study degranulation, cells (1 × 10^6^/mL) were incubated with AO (0.5 µg/mL) at 37 °C, 5% CO_2_ overnight. During incubation, AO was actively accumulated in the cytoplasmic granules, quenched each other, and emitted red fluorescence upon excitation. After washing, cells were challenged with the agents as indicated in pRPMI. As a degranulation reporter, AO in the cytoplasmic granules is released into the culture medium. AO in supernatant is then used to label calf thymus DNA in the detection chamber of the LOAD.

For live cell imaging, the AO-loaded KU-812 cells (5 × 10^4^/mL) were seeded in a glass bottom dish (Ibidi, Germany) coated with polylysine inside and ITO outside. For polylysine coating, polylysine solution (0.1 mg/mL) was placed into the confocal dish for 30 min at room temperature. After washing three times with phosphate buffered saline (PBS), the dish was air-dried for 2 h. Cells were then seeded, and the non-adherent KU-812 cells in suspension were able to stick onto the glass for live cell imaging. To monitor the real-time degranulation, cells in pRPMI medium were scanned with an agron laser (488 nm) with or without stimulation, and the AO green and AO red emissions were recorded separately and simultaneously by confocal microscopy (Fluoview FV100, Olympus, Tokyo, Japan).

For flow cytometry, the AO released from KU-812 cells in the buffer was transferred to another test tube to label the DNA of the fixed HepG2 cells. The fixed cells were prepared by treating HepG2 cells (1 × 10^6^/mL) with 1% paraformaldehyde for 30 min. The AO green fluorescence from the fixed HepG2 cells labeled with the released AO was determined by flow cytometry. Flow cytometric analysis was performed on a FACSCanto flow cytometer (BD Biosciences, San Jose, CA, USA) using WinMDI 2.9 software for data acquisition and analysis.

### 2.3. LOAD Design and Fabrication

The aim of our study was to build a LOAD platform for the BAT. The current design of the disc contains seven identical units. Our LOAD consists of two layers, with the upper PDMS layer housing all microfluidic items, and the lower PMMA (polymethyl methacrylate) layer providing a flat substrate bound to the upper layer for support. The mold of the PDMS with sample inlets, chambers, channels and valves was made with a CNC milling machine. The PDMS positive relief (~2.5 mm thick) was shielded by optical adhesive film (MicroAmp), assembled to the PMMA substrate (3 mm thick) with embedded ITO glasses (size: 10 mm × 18.5 mm; thickness: 0.1 mm; resistance: 200 ohm) and thermistors in a way such that the reaction chambers were located on the heating zone of the ITO glasses. For the fabrication of the ITO heater, two copper electrodes were assembled on the ITO glass with silver paste and dried at 50 °C for 3 h. Subsequently, the ITO heaters were assembled to the machined PMMA substrate by epoxy, followed by the assembly of thermistors with the ITO glasses by copper nano-ball-doped PDMS to improve contacts. For the thermal control, a micro-controller (ATmega2560, Atmel, San Jose, CA, USA) with an A/D converter and a pulse-width-modulator was used. Ten volts were applied to the electrodes, and the maximum current was 50 mA. A desired thermal condition was set and controlled through negative feedback loops. To power the heater in our LOAD, a split core transformer was used, as mentioned in our previous publication [18]. The electrical power was supplied by induction in high-frequency switching mode that was independent of spinning speed in the disc.

Through the use of different spinning speeds, centrifugation force so-generated allowed reagents or cells to pass through different capillary stop valves (V1–V4) and a siphon valve (V5) to the reaction chamber C5 and detection chamber C6, given that the burst speeds for valves V1 to V4 were 425, 550, 800 and 550 rpm, respectively. Operations and liquid manipulations in the LOAD were performed as follows. Firstly, reagents or cells in a buffer (12 μL) were loaded to C1 to C4 through their inlets (C1: calf thymus DNA solution (final concentration 0.5 mg/mL); C2: physiological buffer or patient’s serum; C3: potential allergen, Traditional Chinese Medicine (TCM) or agents for positive control; C4: KU-812 cells loaded with AO (40 × 10^6^/mL). Secondly, centrifugation speed was increased to 650 rpm to allow the sample in C1 to travel to the detection chamber C6, and samples in C2 and C4 to travel to the reaction chamber C5. Speed was kept at 650 rpm for 30 min if sensitization (to allow patient’s IgE to bind to the Fc receptors on basophils) is needed. Thirdly, centrifugation speed was increased to and kept at 1000 rpm for 3 h so that sample in C3 entered the reaction chamber C5. Fourthly, the disc rotation speed was set at 100 rpm for 30 s, and the samples in C5 filled up the siphon valve V5. Lastly, the disc speed was increased up to 1000 rpm again until the end of the assay. Under such a circumstance, the siphon valve V5 opened, and a fixed amount of the supernatant in C5 entered the detection chamber C6. Fluorescence signals were then acquired from the LOAD via a photomultiplier tube.

### 2.4. Statistical Analysis

Results were expressed as mean ± SD of three to four determinations. Data were compared using the Student’s *t* test; *p* values less than 0.05 were considered statistically significant.

## 3. Results and Discussion

### 3.1. BAT Assay with AO-Loaded KU-812 Cells

**Figure 1:**
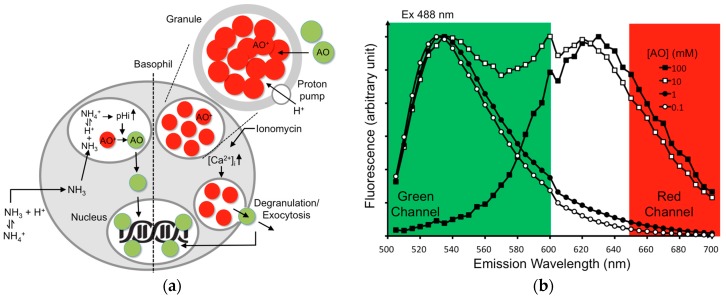
AO are trapped in the granules of basophils and exhibit red shift at high concentrations. Weakly basic AO molecules are selectively accumulated within granules as a result of the intragranular acidic pH. Upon entering the basophilic granules, AO dyes become protonated and are trapped inside the organelles (**a**, right). The resulting high concentrations of AO cause a red shift, thereby giving red fluorescence after excitation at 488 nm. When challenged with NH_4_Cl, NH_4_Cl decomposes into ammonia in solution that passes through membranes easily, accumulating in the basophilic granules and promoting granular alkalinization to release AO (**a**, left). When challenged with ionomycin, the intracellular Ca^2+^ ion level ([Ca^2+^]*_i_*) increases, which triggers degranulation and releases AO externally (**a**, right). The released AO dyes then bind to the nuclear DNA and generate strong green fluorescence (**a**). To demonstrate the phenomenon of red shift, AO was dissolved at the concentration (mM) as indicated (0.1 (open circle), 1 (solid circle), 10 (open square), 100 (solid square)). Emission spectrum of AO at each concentration was scanned from 500 to 700 nm with excitation at 488 nm; emission peak from the solutions of various AO concentrations was normalized to the same level for comparison because the quantum efficiency of the AO red fluorescence is much weaker than that of the green one. High concentrations of AO (≥10 mM) show a red shift (**b**).

KU-812 cells are a basophilic line established from the peripheral blood of a patient with myelogenous leukemia. Apart from carrying human IgE Fc receptors, KU-812 cells are surface non-adherent and do not stick to the channel surface, thus providing good responses in the LOAD. In our system, KU-812 cells were used for the BAT. To reduce the system’s complexity, a fluorescent reporter AO was used as a mimetic in our platform to determine degranulation and exocytosis. Mechanistically, AO enters acidic granular compartments as a weak base in basophils. The uncharged AO dyes inside the granules with low pH are protonated and thus entrapped and sequestered. When accumulates to high concentrations in granules, AO fluoresces red after excitation. Figure 1a is an illustration showing the working principle of using AO as a reporter for degranulation. As can be seen, AO at high concentrations (≥10 mM) exhibits red shift (Figure 1b). On the other hand, when AO binds to DNA molecules, it emits strong green fluorescence (see later part). With these properties, AO is a versatile reporter, able to label acidic granules and nuclear DNA with different color emissions (Figure 1a).

**Figure 2:**
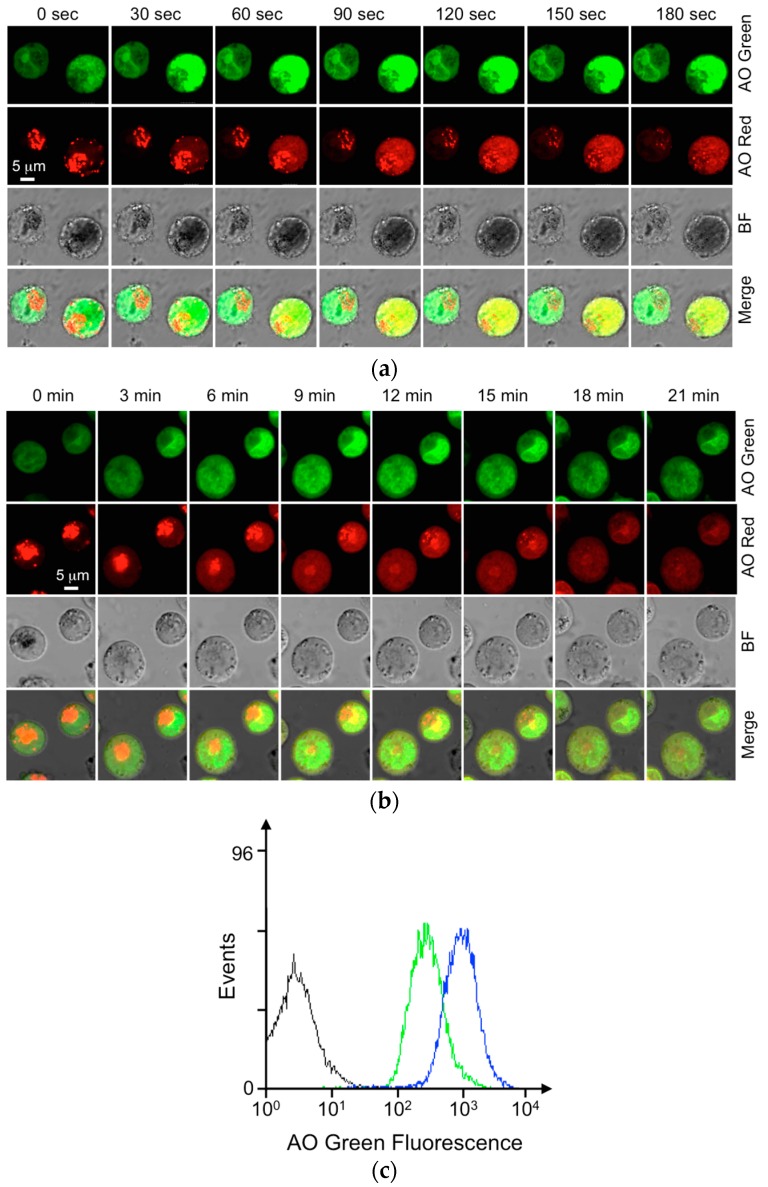
AO was released from the granules of live KU-812 cells by NH_4_Cl and ionomycin with PMA. KU-812 cells (1 × 10^6^/mL) were loaded with AO (0.5 μg/mL) overnight at 37 °C, 5% CO_2_. During incubation, AO was actively accumulated in the acidic granules. After loading, cells in suspension were placed in a confocal dish coated with polylysine. After stabilization, cells were activated with NH_4_Cl (10 mM) (**a**) or ionomycin (1.0 μM) with PMA (20 nM) (**b**) at time zero. Bright field (BF), AO red fluorescence, AO green fluorescence images were acquired from a confocal microscope at the time as indicated. Scale bar represents the cell dimension; after stimulation, AO released in the supernatant was transferred to another Eppendorff to label DNA in the chemically fixed HepG2 cells. Histograms from left to right (*x*-axis in log scale): fixed HepG2 cells in PBS; fixed HepG2 with the supernatant from untreated KU-812 cells; fixed HepG2 with the supernatant from KU-812 cells treated with ionomycin (1.0 μM) and PMA (20 nM) for 20 min at 37 °C, 5% CO_2_ (**c**).

To validate these principles in live cells, we challenged the AO-loaded KU-812 cells with NH_4_Cl and observed the change of the AO red and AO green fluorescence under a confocal microscope using different emission wavelength channels. For the challenge, NH_4_Cl in solution dissociates into ammonia, which diffuses readily across the plasma and internal membranes. Once inside the basophilic granules, the equilibrium NH_4_Cl ⇌ NH_3_ + H^+^ shifts back, pH gradient is disrupted, and the uncharged AO is released from granules into the cytosol (Figure 1a, left panel). Figure 2a shows the effect of NH_4_Cl or granule alkalinization on the release of AO in the live KU-812 cells. As can be seen from the confocal images, AO was selectively concentrated in the cytoplasmic granules and emitted red fluorescence before the challenge. Immediately after the addition of NH_4_Cl, a fall in the AO red fluorescence in the granules was observed with a concomitant increase in the AO green fluorescence in the nuclear region with DNA (Figure 2a). Similar results were obtained when the AO-loaded cells were stimulated with ionomycin and PMA, since basophilic degranulation is a PKC (protein kinase C)-Ca^2+^-dependent reaction (Figure 1a and Figure 2b). Here, ionomycin and PMA are the common activators for the activation of Ca^2+^ and PKC, respectively. Different from the challenge with NH_4_Cl, ionomycin-PMA, a more physiological stimulation, took a longer time to release AO and to label the nuclear DNA (30 s *vs.* 6 min) (Figure 2a,b).

For the measurement of allergic degranulation in live cells, a number of methods have been developed in the last two decades. For example, FITC-dextran [19] and 5-hydroxytryptamine (5-HT) [20] were employed as the degranulation reporter in mast cells and basophils. Yet these methods are technically demanding and labor-intensive. For instance, degranulation was significantly influenced by the duration of cell adhesion in the case of FITC-dextran [19], and a 3-photon set-up was required for the imaging of 5-HT [20].

Loading AO into basophilic granules is another alternative to studying degranulation or exocytosis [21,22]. This BAT assay is cost-effective and simple to use. However, noise was found in the AO assay, as the released AO re-enters cells and labels their own DNA. In light of these issues, we modified the AO assay to report degranulation from the human basophilic KU-812 cells. To increase the signal-to-noise ratio for the readouts, the AO molecules released from KU-812 granules in the supernatant were saved and used to label the DNA of fixed HepG2 cells in another test tube. It is obvious from the flow cytometric analysis that the AO released from the KU-812 cells after ionomycin-PMA treatment was able to generate a stronger green fluorescence with the paraformaldehyde-fixed HepG2 cells when compared to that of the untreated group (Figure 2c). Using this BAT assay, a different degree of degranulation from the control and activated groups can be determined.

**Figure 3:**
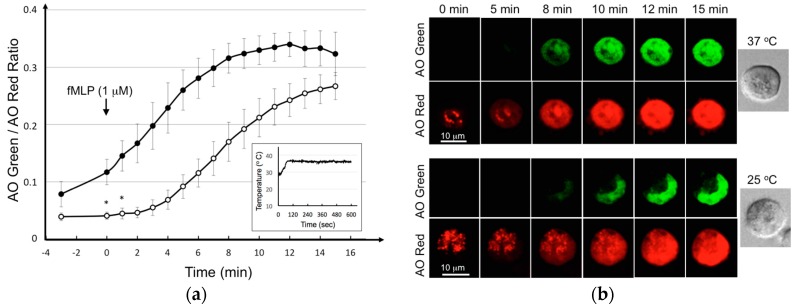
Release of AO from the granules of live KU-812 cells by fMLP at different temperatures. AO-loaded KU-812 cells were prepared for live confocal imaging as described in Figure 2. Cells were then challenged with fMLP (1 μM) at time zero. Bright field, AO red fluorescence, AO green fluorescence images were acquired from a confocal microscope at the time as indicated at 25 or 37 °C in an ITO-coated confocal dish. Experiments were repeated at 25 or 37 °C. The ratio of AO green to AO red from cells were calculated and plotted against time, 25 °C: open circle; 37 °C: solid circle. Results are mean ± SD from 5 single cells in one assay. Results were analyzed by Student’s t-test and the *p*-values less than 0.05 were considered statistically significant. All the data points have a *p* < 0.05 when compared to the corresponding control values except those with * (**a**); scale bar in confocal images represents cell dimension (**b**).

Cellular response in a cell-based assay is strongly affected by the culture environment, especially the temperature. In this study, we tested the utility of using indium tin oxide (ITO) to keep an optimal thermal condition for the BAT. ITO is an optically transparent conductive material that holds great promise for maintaining a constant temperature through feedback control loops in a cultured cell bed outside a cell incubator [23]. As can be seen in the inset of Figure 3a, temperature could be controlled precisely at 37 °C in a culture dish coated with ITO on the external surface. In such condition, KU-812 cells degranulated more vigorously in response to fMLP, a chemotactic peptide triggered basophilic degranulation [24], at 37 °C than that at room temperature (Figure 3b). Experiments were repeated and data were statistically shown in Figure 3a. Obviously, as an optically transparent material, ITO did not appear to affect live cell imaging; as an electrically conductive material, the ITO microheater is a good thermal system for the BAT. Now, this technological know-how enables us to readily translate into the LOAD system.

### 3.2. LOAD Design

**Figure 4:**
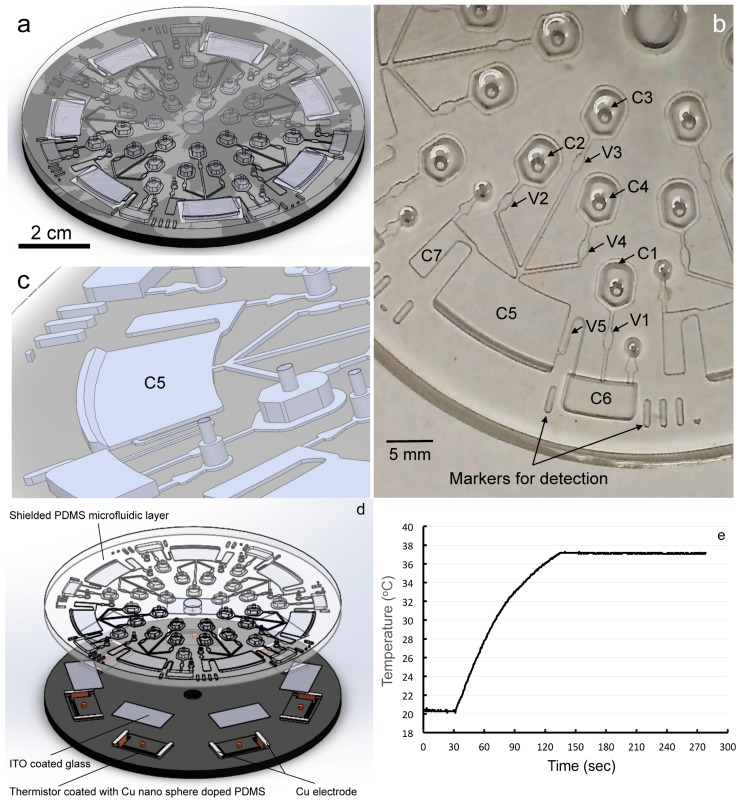
The next generation LOAD for testing allergy. A schematic diagram showing the layout of the LOAD with 7 identical units (**a**); arrangement of chambers, channels, valves and siphon in one unit for testing allergy (**b**); a zoom-in diagram showing the flange in C5 (**c**); assembly of the integrated microfluidic layers with ITO-glass microheaters, electrodes and thermistors is given in panel (**d**); a typical temperature profile in one reaction chamber of the LOAD using an ITO microheater to control temperature at 37 °C (**e**).

Figure 4 shows the design layout of the LOAD for allergy testing. The next generation LOAD is composed of 7 identical units, which can be expanded easily to accommodate more functional units for assays (Figure 4a). As shown, one operation unit contains four storage compartments (C1–C4), one reaction chamber (C5), one detection chamber (C6) and one waste chamber (C7) connected with channels with capillary stop (V1–V4) or siphon (V5) valves (Figure 4b). The analysis process is designed in such a way that samples in the chambers are released sequentially into the next chamber when the spinning speed meets the required burst speed to open the capillary valves.

Practically, the AO-loaded KU-812 cells were first incubated with a buffer alone in C4. Then, cells in C4 and the buffer or the patient’s serum in C2 were spun down to the reaction chamber C5 where they were challenged by potential allergens (from storage chamber C3). In C5, a flange was added at the bottom to increase surface area in the reaction chamber so that cells have a better chance of reacting with stimuli (Figure 4c). If the IgE molecules on the sensitized KU-812 cells were cross-linked by the IgE-specific allergens, the AO would then be released from the granules into extracellular buffer. Subsequently, AO in the supernatant was transferred to the detection chamber C6 through the siphon V5 when the capillary force was larger than the centrifugation force. Now, the AO in the detection chamber C5 labeled the calf thymus DNA in solution in C6. After excitation at 488 nm, green fluorescence from the AO-DNA in C6 can be determined by a PMT. To enhance the signal acquisition process, 2 markers are positioned on the left and right side of the detection chamber to highlight the C6 position. In the case of testing non-IgE type degranulation triggers, the candidate can be injected into C3 (Figure 4b).

Figure 4d shows the layout of our LOAD with the ITO-coated glass microheaters. As can be seen, two layers of microfabricated plates (PDMS and PMMA) were bonded together with the ITO-coated glasses embedded underneath the reaction chambers of the LOAD. Two copper electrodes were placed on the edges of a ITO-glass microheater that was connected to a thermistor. As mentioned, ITO is a conductive transparent material that can generate heat due to its electric resistance when currents flow through it. By using negative feedback loops, the temperature was able to remain at a steady state (37 °C) with a small variation in temperature (±0.15 °C) (Figure 4e). Additionally, it took only 120 s to increase the temperature of the reaction chamber from room temperature to 37 °C (Figure 4e). Collectively, our results show that the ITO microheater is a valuable tool for expanding the utility of our LOAD for BATs with a stable temperature environment. Additionally, it provides a fast, efficient, direct and localized heating facility close to the target zones. This facility is critical for a polymerase chain reaction (PCR) to amplify DNA-based markers with thermal cycling for bio-sensing. Putting the LOAD setup in a temperature-controlled chamber is therefore not a good design, as temperature cannot be cycled in such a fast fashion in a jacket chamber.

### 3.3. Triptolide Suppressed the fMLP-Mediated Degranulation in KU-812 Cells

**Figure 5:**
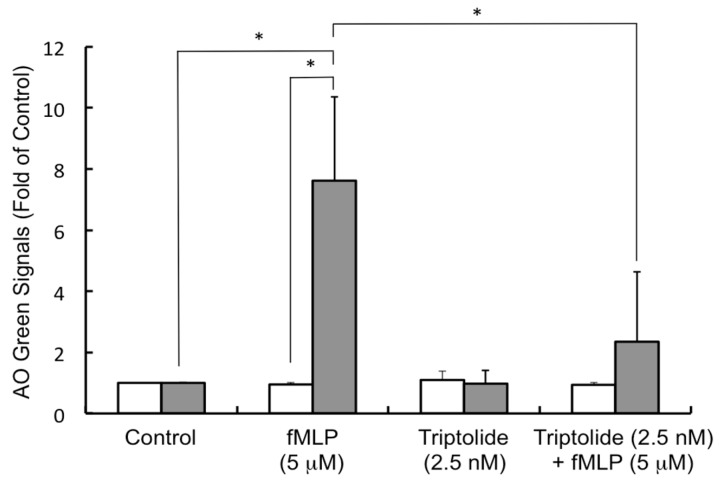
Triptolide suppressed the fMLP-mediated AO release in KU-812 cells. AO-loaded KU-812 cells were prepared for assay as described in Figure 2. Cells were then challenged with medium buffer alone (Control), fMLP (5 μM) or triptolide (2.5 nM) for AO release at room temperature (open bar) or 37 °C (solid bar) in the LOAD. In the last experiment, cells were pretreated with triptolide (2.5 nM) overnight at 37 °C, 5% CO_2_. After washing, the AO-loaded KU-812 cells were challenged with fMLP (5 μM) in the LOAD at room temperature or 37 °C. Signals from test groups are normalized with the control data. Results are mean ± SD (*n* = 4), * *p* < 0.05.

With the establishment of the next generation LOAD, we employed the platform to screen agents from TCM and natural products for the alleviation of allergic symptoms using KU-812 and fMLP as a model. As expected, degranulation was much stronger (7-fold higher) when cells were challenged with fMLP than the medium alone at 37 °C (Figure 5). Contrary to what we had obtained in Figure 3, fMLP could not generate a significant response from KU-812 cells in the LOAD at room temperature (Figure 5). This discrepancy may be due to the low ratio of fMLP molecules per cell in the LOAD set-up. Next, we screened a number of TCMs and flavones, such as triptolide, gliotoxin, gallic acid, luteolin, aplgenin, hesperetin, biochanin-A, naringenin, napthoflavone, for the AO release. Among these agents, only triptolide showed benefits. Other agents did not demonstrate positive effects (data not shown). As shown in Figure 5, triptolide, at concentration of 2.5 nM, did not release the AO from granules at room or body temperature. However, when the KU-812 cells were pre-treated with triptolide (2.5 nM) overnight, it suppressed the fMLP-mediated AO release in a significant manner. Of note, triptolide at this low concentration did not show any cytotoxicity in the KU-812 cells by the MTT assay (data not shown). In the literature, triptolide, as the active substance of TCM *Tripterygium wilfordii* Hook f., has been reported for many years as being used to treat autoimmune disease and inflammation [25]. Now, we show here for the first time that triptolide has an anti-allergy effect. Taken together, this is a good example of how the LOAD can be used, in addition to allergen identification, to screen agents that can alleviate allergy symptoms.

## 4. Conclusions

A LOAD is an emerging powerful tool for allergy testing. In this paper, we highlighted the principles of the KU-812 platform and new developments of our LOAD to screen drugs to relief allergy symptoms. Using our next generation LOAD with an ITO microheater and temperature controller, we identified one TCM herb (triptolide) that can be used to suppress allergic reactions. Looking forward, it is expected that more allergy-blocking agents will be identified using our LOAD for further validation in animal models and clinical trials.
